# On Titular and Other Decorations

**Published:** 1919-01-11

**Authors:** 


### ON TITULAR AND OTHER DECORATIONS.

It is doubtless inevitable that the course and conclusion of a great war should, in a society organised as is the one in which we live, be attended by an enormous increase in the number of titular and other personal decorations. To challenge such a development would be to challenge what is generally accepted as the natural order of things. Many individuals without question have under the stress of national danger rendered great service to the State, and in some instances this service has involved direct personal sacrifice. To grudge a recognition which gives personal pleasure and has, further, a certain professional and social value would seem a churlish thing. Perhaps the possibility of such a recognition does in some instances stir to efforts that otherwise might remain undisplayed, though service so provoked cannot be of the first order. All the same, in its practical exhibition there may be helpfulness to the community. And in any event, at the present stage of human development there is a desire that a visible palm shall be awarded to him who merits it. The difficulty in practice is to command a judgment which will infallibly and invariably appraise this merit. The task is not an easy one in ordinary times, but it becomes raised to a high level when candidates are unusually numerous. From various quarters in recent days have come proposals for altering and improving the machinery of selection, and this is an intimation that the present method excites some degree of discontent. The new proposals may be good or bad, but it is perhaps well to recognise that universal satisfaction in such a matter is not within reach. At the best the choice is that of a fallible judgment acting often on imperfect information and not invariably free from personal partiality or prejudice. Even the decision of a universal suffrage leaves some grumblers.

Where the harm of a very free distribution of decorations arises is in the suggestion that those who escape the shower are thereby marked as below the level of duty. As the ornament becomes more and more frequent it is the absence of it rather than its possession which excites comment. Thus into any organised service, and more or less into society generally, there is introduced the vicious principle that the aim of effort is less the conquest of achievement than the attraction of attention. The smiles of the powers that be can be cultivated by methods of which not all are dignified and worthy, and the search for these is not among the lofty ambitions. To long for the lime light may be a human frailty, but it is not a praiseworthy aim, and the fact that the path of duty is the way to glory is not the best motive for its pursuit. Such are some of the penalties which attend the existing system and there is no clear way of avoiding them. At the present moment there is a very natural desire that those who have deserved well of the country should receive recognition and that in some instances this recognition should have a titular form and quality. We have no opposition to offer to this wish, but what we think might be cultivated with not less earnestness is the resolution that good will shall express itself with even-handed justice. Men who have served their country to their own detriment may be numbered by the thousand and they ought to have the first claim on the State. By all means let great services be recognised by ornaments now deemed appropriate, but it is at least an equal demand that a generous hand shall be kept for the many who, perhaps in humble stations, have given freely to the country and stand to-day permanently handicapped by the sacrifice.

